# Smokeless tobacco and oral potentially malignant disorders in South Asia: a protocol for a systematic review

**DOI:** 10.1186/s13643-016-0320-7

**Published:** 2016-08-24

**Authors:** Zohaib Khan, Sheraz Khan, Lara Christianson, Sara Rehman, Obinna Ekwunife, Florence Samkange-Zeeb

**Affiliations:** 1 Leibniz Institute for Prevention Research and Epidemiology-(BIPS), Achterstrasse 30, 28359 Bremen, Germany; 2 Khyber Medical University, Peshawar, Pakistan; 3 Pakistan Cancer Support Group, Khyber Pakhtunkhwa, Pakistan; 4 Department of Social Welfare and Special Education, Khyber Pakhtunkhwa, Pakistan; 5 Pakistan Paraplegic Centre, Peshawar, Pakistan; 6 Department of Anthropology and Cultural Research, Bremen University, Bremen, Germany; 7 Department of Clinical Pharmacy and Pharmacy Management, Nnamdi Azikiwe University, Awka, Nigeria

**Keywords:** Oral potentially malignant disorders, Smokeless tobacco, Leukoplakia, Submucous fibrosis, Erythroplakia, Paan, Betel quid, Gutkha, South Asia

## Abstract

**Introduction:**

Oral potentially malignant disorders (OPMDs) are chronic lesions or conditions characterized by a potential for malignant transformation. Apart from being possible pre-cursors to oral cancer, OPMDs themselves are usually painful and debilitating conditions having an influence on the quality of life, both in terms of pain and social disability. Smokeless tobacco (SLT) use is considered a major risk factor for OPMDs. SLT use is a culturally and socially acceptable habit in South Asia. According to a recent report, 90 % of the SLT burden of the whole world lies in the South Asian countries of Pakistan, India, Sri Lanka, Bangladesh, Bhutan, Nepal, Afghanistan, and Maldives. This review aims to assess the association between the use of various SLT products in South Asia and risk of OPMDs.

**Methods:**

This review will focus on epidemiological studies on the use of SLT and risk modification for OPMDs, which have been carried out in the human population of South Asian countries. Articles reporting estimates of relative risk, e.g., odds ratio (OR) or relative risk (RR) with their 95 % confidence intervals (CI) for SLT users versus non-users. Articles reporting data from which these effect estimates can be computed will be included in the review. We will search MEDLINE, the Science Citation Index (SCI), Scopus, and the Cumulative Index to Nursing and Allied Health Literature (CINAHL) databases for relevant literature using a combination of keywords and MeSH terms, where applicable. Appropriate sources of gray literature will also be included in the search. The electronic searches will be supplemented by a hand search of the bibliographies of the included articles. The included studies will be assessed for their quality using an established quality assessment tool. All relevant data from the included articles will be recorded in an MS Excel spread sheet and then transferred to Rev Man 5.3 to carry out a meta-analysis. Heterogeneity among the estimates will be assessed through the *I*^2^ statistic. Sensitivity and subgroup analysis will be carried out to see the effects of individual or group of studies on the pooled effect estimate. Results of the review will be reported according to the Preferred Reporting Items for Systematic Reviews and Meta-Analyses (PRISMA) guidelines.

**Discussion:**

This review may have a potential limitation with regard to the designs of the studies included as we expect that most of the included studies will be of the observational types. We will however try to address this issue by conducting sensitivity and subgroup analysis of similar quality studies.

**Systematic review registration:**

PROSPERO CRD42015029705.

**Electronic supplementary material:**

The online version of this article (doi:10.1186/s13643-016-0320-7) contains supplementary material, which is available to authorized users.

## Introduction

Oral potentially malignant disorders (OPMD) are chronic lesions or conditions characterized by a potential for malignant transformation. More specifically, “It is a group of disorders of varying etiologies, usually tobacco; characterized by mutagen associated, spontaneous or hereditary alterations or mutations in the genetic material of oral epithelial cells with or without clinical and histo-morphological alterations that may lead to oral squamous cell carcinoma transformation” [1]. Leukoplakia (LP), erythroplakia (EP), submucous fibrosis (SMF), lichen planus (LP), actinic keratosis (AK), discoid lupus erythematosus (DLE), and palatal lesions among reverse smokers constitute OPMD [2]. Rare, inherited syndromes, e.g., xeroderma pigmentosum and Fanconi’s anemia, and immunodeficiency have also been linked with the development of oral cancer. In addition, patients suffering from chronic Graft Versus Host Disease after stem cell transplantation may also be at a risk of developing oral cancer [3]. It is generally believed that the prevalence of OPMD worldwide varies between 1 and 5 % [4]. The potential for malignant transformation among these conditions vary from less than 1 % to as high as 36 % [5] and is often influenced by the post-diagnosis cessation or continuity of the high risk behaviors, like tobacco and alcohol use, and clinical intervention [6, 7]. LP, EP, and SMF have a higher potential for malignant transformation as compared to other [8]. Apart from being possible pre-cursors to oral cancer, OPMD by themselves are usually painful and debilitating conditions having an influence on the quality of life, both in terms of pain and social disability [9].

Smokeless tobacco (SLT) use is considered as a major risk factor for OPMD [10, 11]. SLT refers to the forms of tobacco which are used without burning the product. It is estimated that SLT contains more than 30 carcinogenic agents [12]. SLT use is a culturally and socially acceptable habit in South Asia [13] according to a recent WHO report 90 % of the SLT burden of the whole world lies in the South Asian countries [14]. South Asia includes Pakistan, India, Sri Lanka, Bangladesh, Bhutan, Nepal, Afghanistan, and Maldives. Different forms of SLT products are used in these countries, often dictated by regional influences [15–18]. The most widely used products include Betel quid or PAAN with tobacco, Gutkha, Naswar, Chaini, Misri, and chewable tobacco leaves. The carcinogenic agents in tobacco act by inducing changes at both genetic level and locally by providing a conducive local environment for hyperplastic transformation of the buccal cells [19].

We conducted pre-review scoping searches to identify the current state of literature on OPMDS, specifically systematic reviews on OPMDs and literature pertaining to the risk of OPMDs associated with the use of SLT. The majority of recent systematic reviews on SLT use have focused on the link between SLT and oral cancer [13, 20, 21], but we identified a knowledge gap with regard to the effects of SLT use and development of OPMDs, particularly in the context of South Asia. We therefore aim to conduct a systematic review on the relationship between SLT and OPMDs to provide evidence which would be beneficial for the scientific community, the tobacco industry, patients, and the general public. It could also help inform the tobacco control policies in South Asia, which have been shown to be underperforming and less orientated when it comes to smokeless tobacco.

## Objectives

### General

The objective of this study is to quantify the risk of developing OPMDs associated with the use of SLT (ever versus never users) by pooling effect estimates from epidemiological studies carried out in South Asia among both males and females of any age group.

### Specific

- To assess the individual risk of different forms of SLT use associated with the development of any OPMD
- To assess the individual risk of development of different OPMDs with the use of SLT
- To assess the risk of developing OPMDs with the use of SLT separately among men and women
- To explore exposure-response relationships in terms of duration and intensity of use of SLT products and the risk of development of OPMDs
- To stratify the risk estimates for individual countries and/or if applicable regions
- To calculate the population attributable fraction (PAF) associated with the use of SLT and development of OPMDs

## Methods

This protocol follows the Preferred Reporting Items for Systematic Reviews and Meta-Analysis Protocols (PRISMA*-*P) 2015, guidelines. The corresponding checklist is provided in Additional file 1.

## Eligibility criteria

### Study design

This review will focus on epidemiological studies investigating the use of SLT and risk modification for OPMDs, which have been carried out in the human population of South Asian countries. Although data generated by experimental research designs, e.g., randomized clinical or community trials, are considered the gold standard, we expect that our search will mostly yield studies with an observational design, due to the inherent ethical issues regarding the potential harm of using SLT. This has been further substantiated through our preliminary scoping searches for the review and a perusal of previous reviews on SLT and oral cancer, where most of the published literature involves observational studies, mostly case-control and cohort designs [13, 20, 21]. However, we will not limit our search to observational designs and will also include experimental studies satisfying our inclusion criteria. Laboratory-based genetic epidemiological studies will not be eligible for inclusion.

### Participants/population

Studies carried out among both males and females irrespective of age, socio-economic, physical, or dental status, who are residents of the countries of South Asia, will be included in the review. For studies to be eligible for the review, the reported cases must have been ascertained as having an OPMD through medical and/or histological records. Studies carried out among expatriate populations of South Asia will not be included, as there may be large differences between tobacco habits and products among the resident populations and the expatriate ones. Studies focusing on animals will not be included.

### Exposure

Studies in which exposure to an SLT product has been ascertained through written records, e.g., medical history, structured interviews, or written self-reports will be included. For the purpose of this review, an “ever” exposed participant is defined as someone who might have used an SLT product at least once in life. Exposure will be quantified in years and daily frequencies for the assessment of the exposure-response relationship between OPMD and SLT. Only the studies reporting daily frequencies and total duration of exposure or reporting data to calculate these will be eligible for the exposure-response analysis. Since SLT products in South Asia are often produced unregulated [22], it is difficult to quantify the intensity of exposure and hence intensity will not be used as an inclusion/exclusion criteria for this review. Studies which exposure of interest is areca nut alone or betel quid without added tobacco, although often investigated together along with other SLT products, will not be eligible for this review as these might contribute to increased heterogeneity because of differences in carcinogenic potential among these and SLT products.

### Comparator(s)/control

For case-control studies, the control group must have included subjects who have no history of OPMDs, irrespective of their use of SLT. Matching for age, sex, and other potential confounders will not be used as inclusion criteria, in order to include maximum studies. The source of controls, i.e., hospital or community-based will not affect the inclusion/exclusion of a study for the review. For cohort studies, the use of a comparator will not be a requirement for inclusion.

### Outcome

Studies which report an OPMD as the, or one of the, primary or secondary outcome/s will be included in the review. Studies where cases were recruited after verification through a medical record or a laboratory report or a clinical examination by a qualified person will be included. Articles reporting estimates of relative risk, e.g., odds ratio (OR) or relative risk (RR) with their 95 % confidence intervals (CI) for SLT users versus non-users (ever versus never) as well as those reporting data from which these effect estimates can be computed will be included in the review. In case there is a potentially eligible study, where effect estimates or data to calculate them is not reported in the manuscript, the authors of the manuscript will be requested to provide the relevant data.

### Follow-up

In order to include maximum studies, the length of follow-up will not be used as an inclusion/exclusion criterion for studies. If applicable, studies having similar lengths of follow-up will be pooled together in a subgroup analysis.

### Setting

Studies will be included irrespective of the study setting, i.e., hospital-based (private and public) or community-based. The only exception will be laboratory based genetic epidemiology studies.

### Language

No filters will be used during the search process and hence studies published in any language will be eligible for inclusion in this review.

### Search methods

We will search MEDLINE, the Science Citation Index (SCI), Scopus, and Cumulative Index to Nursing and Allied Health Literature (CINAHL) databases for relevant literature. A combination of keywords and MeSH terms, where applicable, will be used for the electronic search. A detailed search strategy, developed for this review by an information sciences specialist (LC), is provided in Additional file 2. In order to include all relevant literature, no filters will be used during the electronic search. The electronic search will be supplemented by a hand search of the bibliographies of selected articles and previously published narrative reviews. Efforts will be made to find non-indexed and gray literature pertaining to the topic via an electronic search of regional electronic research repositories especially the World Health Organization’s regional and global Index Medicus, which often contain local literature not indexed with the main stream research indices (search strategy provided as Additional file 3). Internet search engines, e.g., Google Scholar will also be used to identify any relevant literature. Search strategy for these databases is provided in Additional file 4.

### Selection of studies

One author (LC) will create and run the search query in the databases and export the results to reference management software EndNote [23]. Duplicate records will then be removed via the duplicate search function of the reference management software. Two authors (SK and SR) will then independently go through the titles and abstracts of all the records and select the studies relevant to the review. The authors will compare their results with each other and in cases of disagreement, a third author (ZK) will be contacted to resolve the issue, with his/her opinion being decisive. The authors of the original articles may be contacted to clarify issues regarding eligibility. Full texts of the selected studies will be obtained and will be independently screened for inclusion or exclusion in the final review by two authors (SK and SR). The list of selected studies will then be compared and any disagreements will be resolved through a third author (ZK). Finally, the bibliographies of the selected studies will be screened for any relevant studies that might have been missed during the search process. The selection process for the studies will be presented using a flowchart.

### Assessment of the quality of included studies

All selected studies will be assessed for their quality using the National Collaborating Centre for Methods and Tools, McMaster University’s “Effective Public Health Practice Project Quality Assessment Tool for Quantitative Studies” [24]. The authors have previous experience with this tool [13], and in our opinion, it is well suited to the quality assessment of analytical study designs. Studies will be ranked as “strong,” “moderate,” or “weak” based on six parameters, i.e., selection bias, study design, confounding, blinding, data collection methods, and withdrawals and dropouts. In case there are eligible randomized studies, we will use the Cochrane Collaboration tool for assessing the risk of bias, to assess the quality of those studies. Quality assessment will be carried out by (SK and SR) independently. Any disagreements on the quality of the studies will be resolved in the presence of a third author (ZK).

### Data extraction and management

Data regarding study type, publication year, authors name, place of study, sample size, type/s of exposure, exposure frequency and duration, and outcome/s and effect estimates (OR and RR) will be extracted independently by two authors (ZK and SK) and will be recorded onto a pre-designed data extraction spreadsheet (provided in Additional files 5 and 6).

Sample size of the studies where the only outcome is an OPMD will be extracted as such, i.e., the numbers reported in the study. In case there are multiple outcomes, e.g., oral cancer and OPMDs, then the oral cancer cases will be excluded from the sample size and only the cases with OPMD will be reported. Where applicable effect estimates for men and women will be recorded separately in addition to the overall effect estimate.

Based on original reporting in the included studies, the exposure/s (SLT) will be classified during data extraction as Gutkha, Betel quid with tobacco, areca nut with tobacco, chewing tobacco leaves or others. The term “any SLT” will be used for those exposures where the type of SLT is not mentioned in the included studies. Where applicable and depending on the availability of data, a single study might be treated as two or multiple studies if it reports separate risk of OPMD with the use two or more SLT types. Both adjusted (for smoking and alcohol) and crude effect estimates will be recorded.

The outcome for each study will be recorded as one of leukoplakia, erythroplakia, submucous fibrosis, multiple OPMDs or others depending on how they are reported in the included studies. In case the original study has not differentiated between the OPMD types than the term “All OPMD,” will be used to record the outcome for that study. If a study reports separate effect estimates for different types of OPMDs than it will be treated as two or more separate studies corresponding to the related outcome. We will not address co-morbidities and/or secondary outcomes for this review.

Follow up for cohort and trials will be recorded as mean duration of follow up in years.

The individual data recording spreadsheet from each author will then be compared in the presence of a third author (OE), and if there are any differences, the opinion of the third author will be decisive. The third author (OE) can also decide to contact the authors of the included articles to resolve or further clarify issues regarding the data or for additional information. ORs and RRs will be calculated for studies which do not report an OR or RR but have enough data for their calculation. Efforts will be made to calculate an adjusted effect estimate; however, a crude estimate will be used if the available data is insufficient to calculate an adjusted one. The data will then be entered into RevMan 5.3. by ZK.

## Meta, subgroup, and sensitivity analysis

### Data synthesis

Each outcome will be combined and calculated using the statistical software RevMan 5.3, according to the statistical guidelines referenced in the current version of the “Cochrane Handbook for Systematic Reviews of Interventions” [25]. We will pool effect estimates from those included studies to calculate a meta-risk estimate for OPMDs with the use of SLT products using a random effects model. We will use the inverse variance method to account for the potential inconsistencies between the studies. Standard errors for the effect estimates will be calculated from the given confidence intervals after conversion to a log scale. We will carry out quantitative data synthesis if we have one representative study from at least three countries of South Asia. Previous systematic reviews from South Asia on SLT and oral cancer [13, 20, 21] have carried out meta-analysis despite the presence of very high inconsistency *I*^2^ >75 % to provide a meta-risk estimate, because the magnitude of the pooled estimates are often so high that even if we consider the effects of heterogeneity, a causal link between the two can still be suspected. We expect a similar or an even higher magnitude of risk for OPMDs with the use of SLT products in South Asia and therefore will carry out a quantitative synthesis irrespective of the heterogeneity, publication bias and quality of the included studies. The readers though will be informed about the magnitude of the heterogeneity with every pooled effect estimate and the quality of studies, so they could interpret the results in the light of these findings.

The primary analysis will focus on “never versus ever” users of SLT products. We will also calculate individual meta-risk estimates for each subtype of OPMD and also perform a stratified analysis based on the type of SLT products. If feasible other subgroup analysis may include male versus female, stratification by country, as well as the relationship between the magnitude and intensity (dose-response) of the exposure and the outcome. Sensitivity analysis may include inclusion or exclusion of studies with different quality, studies reporting a very high or a very low effect estimate, studies with very large, or very small sample size etc. Reporting bias will be assessed using a visual funnel plot. The analysis will be carried out in RevMan 5.

### Missing data

In case there are missing data, the original authors of the study will be contacted, to obtain the relevant missing data. If missing data cannot be obtained, an imputation method will be used. We will use sensitivity analysis to assess the impact of inclusion of studies which do not report an effect estimate and it has been calculated by the authors or with missing data.

### Assessment of heterogeneity

Heterogeneity will be assessed by visually inspecting forest plots. The *I*^2^ statistic will be calculated for the quantification of inconsistencies and assessment of the effects of heterogeneity in the pooled analysis. A meta-analysis will be performed irrespective of the presence or absence of heterogeneity or a high I^2^ value. Causes of heterogeneity (if present) will be assessed through a subgroup and sensitivity analysis. The cause of heterogeneity may be a difference in sample size between the studies, retrospective versus prospective study designs, difference in background risk among the populations of different countries, representation of females (lower background risk) in the study sample, length of follow up, Inter and intra-country differences between the composition of SLT products, presence or absence of confounders and exposure quantification differences.

The subgroup analysis may involve stratified analysis by country, gender, length of follow-up, adjustment for smoking and alcohol, type of SLT, type of OPMD, and exposure-response categories. Sensitivity analysis may involve dropping of studies with a low quality and studies where crude effect estimates were reported or calculated by the authors. If applicable and depending on the availability of data, meta-regression will be performed to account for the effect of co-exposures and other confounders.

### Assessment of meta-bias(es)

If available, protocols for randomized studies will be accessed and compared with the included study to assess selective reporting of outcomes. However, we expect that analytical studies will pre-dominantly form the core of our included studies and these usually do not have published protocols. We will run both the fixed effect model and the random effects model to assess the possible presence of small sample bias in the included studies. We will further assess publication bias through funnel plots (if the number of available studies is equal to or more than 10). This will be carried out by ZK and OE.

### Confidence in cumulative evidence

The Grading of Recommendations Assessment, Development and Evaluation Working Group methodology will be used to assess the quality of the cumulative evidence. The quality of evidence will be assessed on the basis of risk of bias, consistency, directness, precision, and publication bias by two authors (ZK and FSZ).

### Differences between protocol and review

If applicable, the differences between the published protocol and the review, and the circumstances and reasons, which necessitated these changes will be disclosed to the readers of the review.

## Discussion

This review will identify and synthesize evidence regarding the use of SLT products and the risk of developing OPMDs in the context of South Asia. To the best of our knowledge, this is the first systematic review which will address the issue of SLT use and the risk of development of OPMDs. We therefore, hope that our findings will help inform tobacco control policies in the region as well as empowering health professionals and general public with evidence regarding the deleterious effects of SLT use and the risk of developing OPMDs associated with SLT.

The potential inclusion of pre-dominantly observational studies in the review may raise concerns over the quality of the evidence, as these designs are susceptible to bias(es). However, due to ethical reasons, it is not possible to carry out an experimental study to assess the risks associated with SLT use, and hence, we have to rely on observational data to estimate the risk estimates for OPMDs associated with SLT use. We are aware of this limitation and will aim to minimize the effects of both intra and extra-study factors which might influence the cumulative incidence.

## Additional files

Additional file 1: PRISMA-P 2015 Checklist. (DOCX 29 kb) Additional file 2: PubMed searches, CINAHL search, Science Citation Index search, Scopus search. (DOCX 18 kb) Additional file 3: Search in Global Index Medicus. (DOCX 15 kb) Additional file 4: Google Scholar Search. (DOCX 12 kb) Additional file 5: Characteristics of included studies. (DOCX 14 kb) Additional file 6: Data extraction spreadsheet. (XLSX 11 kb)

## Abbreviations

AK: Actinic keratosis
DLE: Discoid lupus erythematosus
EP: Erythroplakia
LKP: Leukoplakia
OPMD: Oral potentially malignant disorders
OR: Odds ratio
RR: Relative risk
SLT: Smokeless tobacco
SMF: Submucous fibrosis
