# Supplementary material for: Smokeless tobacco and oral potentially malignant disorders in South Asia: a protocol for a systematic review
Source: Syst Rev. 2016 Aug 24;5(1):142. doi: 10.1186/s13643-016-0320-7 (PMC4997723; doi:10.1186/s13643-016-0320-7)
Supplement: Additional file 2: — PubMed searches, CINAHL search, Science Citation Index search, Scopus search. (DOCX 18 kb) [file 13643_2016_320_MOESM2_ESM.docx]

PubMed searches
11.02.2016

Block 1: Smokeless tobacco

| Search name | Search query | Type of search | Results |
| --- | --- | --- | --- |
| 1A | ("Betel quid" OR paan OR Pan OR "Pan Masala" OR "Creamy snuff" OR Gul OR gudhaku OR gutka OR khaini OR khawam OR "Dry snuff" OR mawa OR mishra OR naskar OR "Red tooth powder" OR tuibur OR zarqa OR "smokeless tobacco") | keyword | 98.001 |
| 1B | smokeless tobacco (*) | MeSH  major & sub-terms | 3.047 |
| 1 | 1A OR 1B |  | 98.001 |

Block 2: South Asia

| Search name | Search query | Type of search | Results |
| --- | --- | --- | --- |
| 2A | (Afghanistan OR Bangladesh OR Bhutan OR India OR Maldives OR Nepal OR Pakistan OR Sri Lanka OR Iran OR "south* asia*") | keyword | 525.381 |
| 2B | Asia, Western (*) | MeSH  major & sub-terms | 206.521 |
| 2 | 2A OR 2B |  | 604.243 |

(*) MeSH: Asia, Western
The geographical designation for the countries of the Middle East (Afghanistan, Bahrain, Iran, Iraq, Israel, Jordan, Kuwait, Lebanon, Oman, Qatar, Saudi Arabia, Syria, Turkey, United Arab Emirates, Yemen) and the countries Bangladesh, Bhutan, India, Nepal, Pakistan, and Sri Lanka.

Block 3: Oral potentially malignant disease

| Search name | Search query | Type of search | Results |
| --- | --- | --- | --- |
| 3A | ("Palatal lesions" OR "Lichen planus" OR "Discoid lupus erythematosus" OR "Oral Potential* Malignant" OR precancerous OR Leukoplakia OR "Submucous fibrosis" OR Erythroplakia OR "Actinic keratosis" OR OPMD) | keyword | 49.434 |
| 3B | Mouth Diseases (*) | MeSH  major & sub-terms | 258.503 |
| 3 | 3A OR 3B |  | 297.982 |

(*) MeSH: Mouth Diseases: <http://www.ncbi.nlm.nih.gov/mesh/?term=Mouth+Diseases>

# Summary & results

| Search name (Saved in PubMed & EndNote) | Results |
| --- | --- |
| 1 AND 2 AND 3 | 560 |

CINAHL search
11.02.2016

| Search name | Search query | Type of search | Results |
| --- | --- | --- | --- |
| 1 | ("Betel quid" OR paan OR Pan OR "Pan Masala" OR "Creamy snuff" OR Gul OR gudhaku OR gutka OR khaini OR khawam OR "Dry snuff" OR mawa OR mishra OR naskar OR "Red tooth powder" OR tuibur OR zarqa OR "smokeless tobacco") | keyword | 1.912 |
| 2 | ("Palatal lesions" OR "Lichen planus" OR "Discoid lupus erythematosus" OR "Oral Potential* Malignant" OR precancerous OR Leukoplakia OR "Submucous fibrosis" OR Erythroplakia OR "Actinic keratosis" OR OPMD) | keyword | 2.470 |
| 3 | (Afghanistan OR Bangladesh OR Bhutan OR India OR Maldives OR Nepal OR Pakistan OR Sri Lanka OR Iran OR "south* asia*") | keyword | 24.929 |
| 4 | 1 AND 2 AND 3 |  | 11 |

Science Citation Index search
11.02.2016

| Search name | Search query | Type of search | Results |
| --- | --- | --- | --- |
| 1 | ("Betel quid" OR paan OR Pan OR "Pan Masala" OR "Creamy snuff" OR Gul OR gudhaku OR gutka OR khaini OR khawam OR "Dry snuff" OR mawa OR mishra OR naskar OR "Red tooth powder" OR tuibur OR zarqa OR "smokeless tobacco") | keyword | 44.509 |
| 2 | ("Palatal lesions" OR "Lichen planus" OR "Discoid lupus erythematosus" OR "Oral Potential* Malignant" OR precancerous OR Leukoplakia OR "Submucous fibrosis" OR Erythroplakia OR "Actinic keratosis" OR OPMD) | keyword | 14.486 |
| 3 | (Afghanistan OR Bangladesh OR Bhutan OR India OR Maldives OR Nepal OR Pakistan OR Sri Lanka OR Iran OR "south* asia*") | keyword | 199.541 |
| 4 | 1 AND 2 AND 3 |  | 110 |

Scopus search
15.02.2016

| Search name | Search query | Type of search | Results |
| --- | --- | --- | --- |
| 1 | ("Betel quid" OR paan OR Pan OR "Pan Masala" OR "Creamy snuff" OR Gul OR gudhaku OR gutka OR khaini OR khawam OR "Dry snuff" OR mawa OR mishra OR naskar OR "Red tooth powder" OR tuibur OR zarqa OR "smokeless tobacco") | Title-abstract-keyword | 80.139 |
| 2 | ("Palatal lesions" OR "Lichen planus" OR "Discoid lupus erythematosus" OR "Oral Potential* Malignant" OR precancerous OR Leukoplakia OR "Submucous fibrosis" OR Erythroplakia OR "Actinic keratosis" OR OPMD) | Title-abstract-keyword | 56.376 |
| 3 | (Afghanistan OR Bangladesh OR Bhutan OR India OR Maldives OR Nepal OR Pakistan OR Sri Lanka OR Iran OR "south* asia*") | Title-abstract-keyword | 47.491 |
| 4 | 1 AND 2 AND 3 |  | 31 |

| Search name | Search query | Type of search | Results |
| --- | --- | --- | --- |
| 1 | ("Betel quid" OR paan OR Pan OR "Pan Masala" OR "Creamy snuff" OR Gul OR gudhaku OR gutka OR khaini OR khawam OR "Dry snuff" OR mawa OR mishra OR naskar OR "Red tooth powder" OR tuibur OR zarqa OR "smokeless tobacco") | All fields | 1.916.870 |
| 2 | ("Palatal lesions" OR "Lichen planus" OR "Discoid lupus erythematosus" OR "Oral Potential* Malignant" OR precancerous OR Leukoplakia OR "Submucous fibrosis" OR Erythroplakia OR "Actinic keratosis" OR OPMD) | All fields | 94.903 |
| 3 | (Afghanistan OR Bangladesh OR Bhutan OR India OR Maldives OR Nepal OR Pakistan OR Sri Lanka OR Iran OR "south* asia*") | All fields | 245.516 |
| 4 | 1 AND 2 AND 3 |  | 540 |
