# Supplementary material for: Smokeless tobacco and oral potentially malignant disorders in South Asia: a protocol for a systematic review
Source: Syst Rev. 2016 Aug 24;5(1):142. doi: 10.1186/s13643-016-0320-7 (PMC4997723; doi:10.1186/s13643-016-0320-7)
Supplement: Additional file 3: — Search in Global Index Medicus. (DOCX 15 kb) [file 13643_2016_320_MOESM3_ESM.docx]

**Search in Global Index Medicus^[[1]](#footnote-1)^
Search conducted 10.03.2016**
This resource is a repository for scientific and technical literature which includes the following regional indexes: AIM (AFRO), LILACS (AMRO/PAHO), IMEMR (EMRO), IMSEAR (SEARO), WPRIM (WPRO), Global Index Regional Indexes, MEDLINE, SciELO as well as IRIS, the WHO institutional repository.

Search:

(tw:((afghanistan OR bangladesh OR bhutan OR india OR maldives OR nepal OR pakistan OR sri lanka OR iran OR "south* asia*"))) AND (tw:(("Betel quid" OR paan OR pan OR "Pan Masala" OR "Creamy snuff" OR gul OR gudhaku OR gutka OR khaini OR khawam OR "Dry snuff" OR mawa OR mishra OR naskar OR "Red tooth powder" OR tuibur OR zarqa OR "smokeless tobacco"))) AND (tw:(("Palatal lesions" OR "Lichen planus" OR "Discoid lupus erythematosus" OR "Oral Potential* Malignant" OR precancerous OR leukoplakia OR "Submucous fibrosis" OR erythroplakia OR "Actinic keratosis" OR opmd))) AND (instance:"ghl")
134 results

(tw:((afghanistan OR bangladesh OR bhutan OR india OR maldives OR nepal OR pakistan OR sri lanka OR iran OR "south* asia*"))) AND (tw:(("Betel quid" OR paan OR pan OR "Pan Masala" OR "Creamy snuff" OR gul OR gudhaku OR gutka OR khaini OR khawam OR "Dry snuff" OR mawa OR mishra OR naskar OR "Red tooth powder" OR tuibur OR zarqa OR "smokeless tobacco"))) AND (tw:(("Palatal lesions" OR "Lichen planus" OR "Discoid lupus erythematosus" OR "Oral Potential* Malignant" OR precancerous OR leukoplakia OR "Submucous fibrosis" OR erythroplakia OR "Actinic keratosis" OR opmd))) AND (instance:"ghl") AND ( db:("IMSEAR" OR "LILACS"))
14 results, unique - > all hits from Medline excluded, these hits came from either IMSEAR or LILACS.

1. About the Global Index Medicus, formerly known as the Global Health Library: <http://www.globalhealthlibrary.net/php/level.php?lang=en&component=19&item=1> [↑](#footnote-ref-1)
