# Supplementary material for: Smokeless tobacco and oral potentially malignant disorders in South Asia: a protocol for a systematic review
Source: Syst Rev. 2016 Aug 24;5(1):142. doi: 10.1186/s13643-016-0320-7 (PMC4997723; doi:10.1186/s13643-016-0320-7)
Supplement: Additional file 4: — Google Scholar Search. (DOCX 12 kb) [file 13643_2016_320_MOESM4_ESM.docx]

**Google Scholar Search, run 10.03.2016**

intitle:“smokeless tobacco” AND (Afghanistan OR Bangladesh OR Bhutan OR India OR Maldives OR Nepal OR Pakistan OR Sri Lanka OR Iran OR Asia) AND ("Oral Potential Malignant" OR "Oral Potentially Malignant" OR precancerous OR OPMD)
**55**

**Discarded searches:**

intitle:“smokeless tobacco” AND (Afghanistan OR Bangladesh OR Bhutan OR India OR Maldives OR Nepal OR Pakistan OR Sri Lanka OR Iran OR Asia) AND ( "Oral Potential Malignant" OR precancerous OR OPMD)
**109**

intitle:“smokeless tobacco” AND (Afghanistan OR Bangladesh OR Bhutan OR India OR Maldives OR Nepal OR Pakistan OR Sri Lanka OR Iran OR Asia) AND ( "Oral Potentially Malignant" OR precancerous OR OPMD)
**65**

intitle:“smokeless tobacco” AND (Afghanistan OR Bangladesh OR Bhutan OR India OR Maldives OR Nepal OR Pakistan OR Sri Lanka OR Iran OR Asia) AND intitle: ("Palatal lesions" OR "Lichen planus" OR "Discoid lupus erythematosus" OR "Oral Potential Malignant" OR precancerous OR Leukoplakia OR "Submucous fibrosis" OR Erythroplakia OR "Actinic keratosis" OR OPMD)
**Too long doesn’t work**

“smokeless tobacco” AND (Afghanistan OR Bangladesh OR Bhutan OR India OR Maldives OR Nepal OR Pakistan OR Sri Lanka OR Iran OR Asia) AND ("Oral Potential Malignant" OR "Oral Potentially Malignant" OR precancerous OR Precancer OR OPMD)
**1.100**
