# Supplementary material for: Smokeless tobacco and oral potentially malignant disorders in South Asia: a protocol for a systematic review
Source: Syst Rev. 2016 Aug 24;5(1):142. doi: 10.1186/s13643-016-0320-7 (PMC4997723; doi:10.1186/s13643-016-0320-7)
Supplement: Additional file 5: — Characteristics of included studies. (DOCX 14 kb) [file 13643_2016_320_MOESM5_ESM.docx]

| **Title** | **Date** | **Author/s** | **Study location** | **Study Type** | **No of cases (n1)** | **No of controls (n2)** | **Outcome** | **Exposure*** | **Quality** |
| --- | --- | --- | --- | --- | --- | --- | --- | --- | --- |
|  |  |  |  |  |  |  |  |  |  |
|  |  |  |  |  |  |  |  |  |  |
|  |  |  |  |  |  |  |  |  |  |
|  |  |  |  |  |  |  |  |  |  |
|  |  |  |  |  |  |  |  |  |  |
|  |  |  |  |  |  |  |  |  |  |
|  |  |  |  |  |  |  |  |  |  |
|  |  |  |  |  |  |  |  |  |  |
|  |  |  |  |  |  |  |  |  |  |
|  |  |  |  |  |  |  |  |  |  |
|  |  |  |  |  |  |  |  |  |  |
|  |  |  |  |  |  |  |  |  |  |
|  |  |  |  |  |  |  |  |  |  |
|  |  |  |  |  |  |  |  |  |  |
|  |  |  |  |  |  |  |  |  |  |
|  |  |  |  |  |  |  |  |  |  |
|  |  |  |  |  |  |  |  |  |  |

**Supplementary File 5. Characteristics of included studies**.
